# A novel method for the identification and quantification of N6-methyladenosine motifs in RNA transcripts

**DOI:** 10.1007/s11033-026-12270-3

**Published:** 2026-07-08

**Authors:** Neha Choudhari, H. S. Anirudh Srinivas, Praneeth Sai Tadepalli, Rounak Roy, Souvik Dey

**Affiliations:** Department of Biotherapeutics Research, Manipal Academy of Higher Education, Manipal , India

**Keywords:** m^6^A-methylation, m^6^A-motif prediction, Probe-based ELISA, mRNA stability, Tumor suppressor gene, Early cancer detection

## Abstract

**Background:**

N6-methyladenosine profiles of mRNA transcripts regulate their translocation from the nucleus to the cytosol, stability, and translational efficiency; hence, they have been implicated in gene expression and disease progression. The m^6^A-methylation is widely associated with various cancers and neurological, cardiovascular, and developmental disorders, which demand early diagnosis. A robust m^6^A-motif prediction is necessary to enable us to identify the regulatory nucleic acid sequences that determine mRNA fate in normal and diseased conditions.

**Methods and Results:**

We have developed a transcript-aware computational pipeline, termed *m*^*6*^*A* *Functional Index in Transcription* (m^6^A-FINDiT), that can identify potential m^6^A sites on mRNA transcripts, considering molecular intricacies associated with their secondary structure. This tool can separately identify m^6^A motifs within the coding sequences as well as in non-translatable regions, i.e., 5’UTR and 3’UTR, of mRNA transcripts. Parallelly, another technique was developed that quantifies specific m^6^A methylation motifs through a probe-based ELISA process, MAQ-G. This second method successfully validated the N⁶-methyladenosine motifs predicted by the initially developed motif-finder program.

**Conclusion:**

This integrated m^6^A-FINDiT and MAQ-G, coupled with a real-time qPCR assay, could correlate the methylation profiles of N6-methyladenosine motifs with the expression and stability contours of a gene. To establish the physiological implications of these techniques, we chose three tumour-suppressor genes, viz., *IRF8*, *RB1*, and *TP53* mRNA transcripts, which may undergo m^6^A methylation at certain DRACH motifs. The m^6^A-FINDiT pipeline could successfully predict the specific m^6^A motifs, and the MAQ-G confirmed the methylation profile of the latter. These duo techniques hold potential for use in clinical settings for early cancer detection.

**Supplementary Information:**

The online version contains supplementary material available at 10.1007/s11033-026-12270-3.

## Introduction

The *N6-methyladenosine* (m^6^A) modification found in RNA determines its stability, ability for translocation from the cell nucleus to cytosol, alternative splicing, and translational efficiency [1]. Around 80% of the m^6^A additions happen in mRNA, making it a crucial modification for the regulation of gene expression. m^6^A methylation levels are therefore significant to understand the mRNA regulatory function in various disorders [2]. It can also be an indicator to assess the translational regulation of protein expression involved in cellular development and differentiation. The methyl group is installed on certain adenosine residues of mRNA by the methyltransferases such as METTL3 and METTL14, along with other components constituting the “writers’ complex” [3, 4]. The reversal of methylation is performed by the demethylases or “erasers” such as Fat Mass and Obesity-associated protein (FTO) and AlkB Homolog 5, RNA Demethylase (ALKBH5) [5]. The YT521-B homology (YTH) domain-containing proteins and insulin-like growth factor 2 mRNA-binding proteins (IGF2BP), also called “reader proteins,” identify these m^6^A residues on mRNA transcripts and regulate downstream RNA processing [1]. Disruption of the function of these proteins, leading to abnormal m^6^A levels, has been reported in obesity-associated disorders, neoplastic growth, neurological disorders, and infertility-related conditions [6–11]. The functional efficacy of these motifs hinges on their accessibility within the RNA secondary structure, with single-stranded regions, such as loops or bulges, being more responsive to modification than double-stranded regions like stems [12]. This structural dependency arises because methyltransferases require unobstructed access to the adenosine’s N6 position, which is hindered by base-pairing in double-stranded conformations. A crucial element necessary for methylation is the methylation consensus motif, typically characterized by the DRACH motif in mammalian cells (D = A/G/U, R = A/G, H = A/C/U).

Computational identification of DRACH motifs is critical for predicting m^6^A modification sites. However, many existing tools rely solely on sequence-based detection, overlooking the structural context that governs motif accessibility to methyltransferases. Sequence-based methods frequently result in overpredictions, such as motifs in paired regions that are improbable to undergo modification, whereas machine learning techniques may exhibit limited generalizability owing to training on specific datasets [13]. To address these limitations, we developed a structure-aware computational pipeline: *m*^*6*^*A Functional Index in Transcription*, acronymized as m^6^A-FINDiT, that integrates genomic annotations, mRNA sequence reconstruction, and secondary structure prediction to identify DRACH motifs in unpaired regions of mRNA transcripts. A systematic comparison of our m^6^A-FINDiT pipeline with currently available m^6^A prediction tools (SRAMP [14], DeepM6ASeq [15], WHISTLE [13], HSM6AP [16], m6Aboost [17], DeepM6ASeq-EL [18], CLSM6A [19], and DeepSRAMP [20]) is provided in Supplemental Table 1. This pipeline leverages high-quality genomic resources and minimum free energy (MFE) models to enhance the specificity of m^6^A site prediction, offering a robust tool for studying post-transcriptional regulation across diverse genes and species.

These predicted m^6^Amotifs can be further validated for m^6^A modification through m^6^A RNA immunoprecipitation using a highly sensitive m^6^A antibody [21–25]. Currently used ELISA assays and commercial m^6^A quantification kits enable rapid estimation of global m^6^A abundance but do not provide transcript- or sequence-specific information [26–29]. They also become economically limiting in studies involving large sample sizes [30, 31]. Recent advances in m^6^A detection technologies have considerably improved the ability to profile RNA methylation. However, these existing approaches still present practical and technical limitations. Transcriptome-wide methods such as MeRIP-seq/m^6^A-seq have enabled broad mapping of m^6^A modifications across the transcriptome [32, 33] but are constrained by relatively low resolution, antibody-associated bias, higher RNA input requirements, and the need for next-generation sequencing infrastructure [31]. Higher-resolution approaches, including miCLIP, SCARLET, SELECT, DART-seq, GLORI, and enzyme-based methods such as MAZTER-seq, enable site- and transcript-specific detection of m^6^A modifications. However, these methods involve complex workflows and require specialized reagents, recombinant proteins, or motif-dependent detection strategies [30]. Sequencing-based approaches depend on deep sequencing to obtain sufficient coverage and statistical significance, making them expensive and less practical for routine laboratory use [34]. Limited coverage and technical variability may also increase false-positive and false-negative detection rates and affect reproducibility across studies [34–37]. To address these limitations, we have developed a cost-effective, ELISA-based colorimetric quantification method termed *Methylation6A Quantification for Genes* (MAQ-G), incorporating complementary capture oligonucleotides (CCOs) designed to target m^6^A-binding motifs in mRNA transcripts identified through the m^6^A-FINDiT pipeline. This technique is ultra-sensitive, enabling targeted detection of m^6^A-modified RNA via complementary capture ssDNA, thereby selectively enriching specific m^6^A-containing transcripts without the need for recombinant RNA-binding proteins or motif-specific antibodies. MAQ-G utilizes a universal m^6^A-specific antibody, making the approach more flexible and economically sustainable across different target transcripts. The method is highly sensitive and can detect RNA inputs as low as 100 pg. This increased sensitivity and sequence specificity expand the feasibility of m^6^A analysis in limited biological samples and support broader application of transcript-specific epitranscriptomic studies and high-throughput research.

## Materials and methods

### Genomic and annotation resources

The pipeline utilized the GENCODE annotation to obtain transcript and exon coordinates for the target gene, ensuring comprehensive inclusion of all isoforms. The primary assembly genome served as the reference for sequence extraction, maintaining consistency with the annotation data. These high-quality resources formed a reliable basis for transcript reconstruction and motif analysis, adaptable to other species or genomic assemblies as required.

### Development of computational pipeline- m^6^A-FINDiT

The pipeline processed mRNA transcripts through a multi-step workflow designed to detect DRACH motifs in structurally accessible regions:

i. Transcript Annotation and mRNA Reconstruction:

Transcripts were identified from the GENCODE annotation, encompassing both protein-coding and non-coding isoforms. Exon coordinates were extracted, respecting strand orientation, to define the spliced mRNA structure for each transcript.

mRNA sequences were reconstructed by retrieving exon sequences from the species-specific genome reference assemblies and concatenating them in the correct order. This comprehensive approach ensured a complete representation of each transcript, mitigating artifacts from analyzing isolated genomic regions.

ii. Secondary Structure Prediction:

RNA secondary structures were predicted using RNAfold, a component of the ViennaRNA package, which employed an MFE-based algorithm optimized to produce stable conformations with minimal complex base-pairing patterns [38, 39]. RNAfold generated specific notations for unpaired bases and paired bases, which helped in distinguishing single-stranded from double-stranded regions.

iii. DRACH Motif Identification and Accessibility Filtering:

mRNA sequences were scanned for the 18 unique DRACH motifs, defined by the consensus sequence formula D-R-A-C-H, where D represents A, G, or U; R denotes A or G; A is adenosine (the m^6^A modification site); C is cytosine; and H indicates A, C, or U. This formula yields 18 possible motif variants (e.g., AAACA, AGACT, GGACU) due to the combinatorial permutations of variable bases (D, R, H) [40].

Motifs were adapted for genomic DNA by substituting T for U to align with the reference genome, ensuring all variants were captured based on their sequence composition. A filtering mechanism retained only motifs located in unpaired regions, as indicated by the dot-bracket notation from RNAfold, where all five bases of the motif are single-stranded. This prioritization enhanced the biological relevance of motifs, as unpaired regions were more accessible to methyltransferases [12]. A complete Indian patent (Application No. 202541116970 A) was filed for the methodology [41]. m^6^A-FINDiT was applied to Interferon regulatory factor 8 (*IRF8)*, Retinoblastoma 1 (*RB1*) and Tumor protein 53 (*TP53*) in both human and mouse orthologs to demonstrate generalizability.

### Cell treatment and Western blot

The MOLT-3, EL4 and A549 cell lines, were obtained from the National Centre for Cell Science (Pune, India) and were treated with 2.5 µM/5 µM FB23-2 inhibitor (cat. #SML2694-5 mg, Sigma, USA) for 24 h. MOLT-3 protein extracts were prepared using the 1X RIPA buffer and quantified following the BCA method. Approximately 40 µg of protein was analyzed by SDS-PAGE and transferred to a PVDF membrane. The membrane was blocked with 5% non-fat dry milk (NFDM) in TBST for 1 h at room temperature, then incubated overnight at 4 °C with FTO primary antibody (1:2000) (cat. #27226-1-AP, Proteintech) in 3% non-fat dry milk. Afterwards, it was probed with an HRP-conjugated secondary antibody and visualized using enhanced chemiluminescence (ECL).

### RNA extraction and quantitative PCR

Total RNA was extracted from MOLT-3, EL4 (Adult T cell acute lymphoblastic leukemia cell line) and A549 (Human alveolar basal epithelial cell line) cells using RNAiso Plus (Takara, Japan) following the manufacturer’s protocol. The RNA concentration was measured with a Synergy H1 microplate reader (BioTek, Winooski, VT, USA). For Reverse transcription, RNA (100 ng) was converted to cDNA using oligo(dT) primers. The gene expression analysis of *IRF8*, *RB1* and *TP53* was performed utilising SYBR Premix Ex Taq II (Takara, Japan) on QuantStudio 5 real-time PCR. qPCR was performed with 100 ng of template cDNA. The forward and reverse primer sequences used for qPCR were the following: 

*IRF8* (Human) - forward primer: 5’-AGGTCTTCGACACCAGCCAGTT-3’, reverse primer: 5’-GCACGAGAATGAGTTTGGAGCG-3’; 

*TP53* (Human) - forward primer: 5’-CCTCAGCATCTTATCCGAGTGG-3’, reverse primer: 5’-TGGATGGTGGTACAGTCAGAGC-3’;

*RB1* (Human) - forward primer: 5’-CAGAAGGTCTGCCAACACCAAC-3’, reverse primer: 5’-TTGAGAACACCGTCGCTGTTAC-3’;

*Rb1* (Mouse) - forward primer: 5’-CCTTGAACCTGCTTGTCCTCTC-3’, reverse primer: 5’-CTGAGGCTGCTTGTGTCTCTGT-3’.

### MAQ-G technique

The study focused on validating transcript-specific RNA motifs accessible for m^6^A modification using an ELISA-based detection method (MAQ-G). Complementary capture oligonucleotides (CCOs) were designed for these motifs, and a full Indian patent (Application No. 202441095672 A)= was filed for the methodology [42]. To prepare high-binding affinity 96-well plates (PerkinElmer, cat. #6005600) for nucleic acid binding, 0.01% poly-L-lysine was added and incubated at room temperature for 2 h. DNA oligos complementary to the m^6^A motifs were added to the coated wells and incubated at 37 °C for 1.5 h. The wells were then blocked using 1% BSA in 0.05% TBST at room temperature for 1 h. Total RNA, diluted in Tris-buffer saline, was added and incubated at 37 °C for 90 min.

Controls included an N6-methylated oligo with a DRACH motif (positive control), DEPC-treated water or TE buffer (blank), and a non-methylated version of the sequence (negative control). Unbound nucleic acids were washed out with 0.05% TBST. The wells were incubated with anti-m^6^A mouse primary antibody (Abcam, #ab208577) in 1% BSA for 1 hour at room temperature. After multiple washes, an HRP-conjugated secondary antibody was added and incubated for 30 minutes. Following five rounds of washing, a colorimetric reaction was initiated with tetramethylbenzidine (TMB)/H2O2 until a blue color developed. The reaction was stopped with 2 N H₂SO₄, turning the solution yellow, and absorbance was measured at 450 nm. The generic CCO sequence used is as follows: 5’-ACAGGCAAGTCCAACACGAACAGGCAAC-3’. For gene-specific m^6^A estimation, the top two CCOs identified m^6^A-FINDiT were used for each gene to target specific DRACH motifs. The following probes were used to assess m^6^A levels in RNA from control and FB23-2-treated cells:

*IRF8* (Human) - probe1: 5’-CTGAATGACAAGTCTTTGGAAATGA-3’, target motif: AGACT; probe 2: 5’-AAAACAACCTTGTTTTCACAAGTTG-3’, target motif: AAACA.

*TP53* (Human) - probe 1: 5’-AGGCAAGGAGTGTCTTGCTGAGAGA-3’, target motif: AGACA; probe 2: 5’-CTGCTTGTCCTGTTTGGCTGAGGTA-3’, target motif: AAACA.

*RB1* (Human) - probe 1: 5’-TCACCATGGCGGTCATCAGGCTCAG-3’, target motif: TGACC; probe 2: 5’-GATCACTTGCTGTCCGCAATAATAT-3’, target motif: GGACA.

*Rb1* (Mouse) - probe 1: 5’-GATCACTGGCTGTCCTCAGTAATAT-3’, target motif: GGACA; probe 2: 5’-TTCCAAAGCCGGTCCTGGGCCCTGC-3’, target motif: GGACC.

### Immunofluorescence studies

Immunofluorescence staining was done on control and FB23-2 inhibitor-treated MOLT-3, EL4 and A549 cells to compare methylation status. A549 adherent cells were seeded on poly-L-lysine-coated coverslips and treated accordingly prior to fixation. MOLT-3 and EL4 suspension cells were fixed for 10 min of 4% paraformaldehyde at room temperature. After permeabilization on ice with 0.1% Triton X-100 in calcium- and magnesium-free Dulbecco’s phosphate-buffered saline (DPBS) for 10 min, cells were resuspended in DPBS, fixed, and permeabilized cells adhered to poly-L-lysine-coated coverslips for 1 h at room temperature. Non-adherent cells were eliminated by washing coverslips three times with 1× TBST (0.5% Tween-20) for 5 min each. The cells were blocked with 5% BSA in TBST for 1 h at room temperature. Following blocking, cells were incubated overnight at 4 °C with an anti-N6-methyladenosine (m^6^A) antibody (Abcam, ab208577) diluted 1:200 in 3% BSA prepared in TBST. Subsequently, Alexa Fluor™ 568-conjugated secondary antibody (Invitrogen) was used at a 1:1000 dilution for 1 h at room temperature. After thorough washing, coverslips were mounted using ProLong™ Diamond Antifade Mountant (Invitrogen). Images were obtained using a confocal fluorescence microscope (Leica TCS SP5) and analyzed using Leica Application Suite 4.0.

### Statistical analysis

All statistical analyses were performed using the one-way ANOVA and Student’s unpaired t-test by using GraphPad Prism 8.0.1 (GraphPad Software Inc.). The normality of the distribution of the obtained data was analyzed by Q-Q plots.

## Results

### Development of a comprehensive workflow for the identification and quantification of N6-methyladenosine motifs in mRNA transcripts

**Fig. 1:**
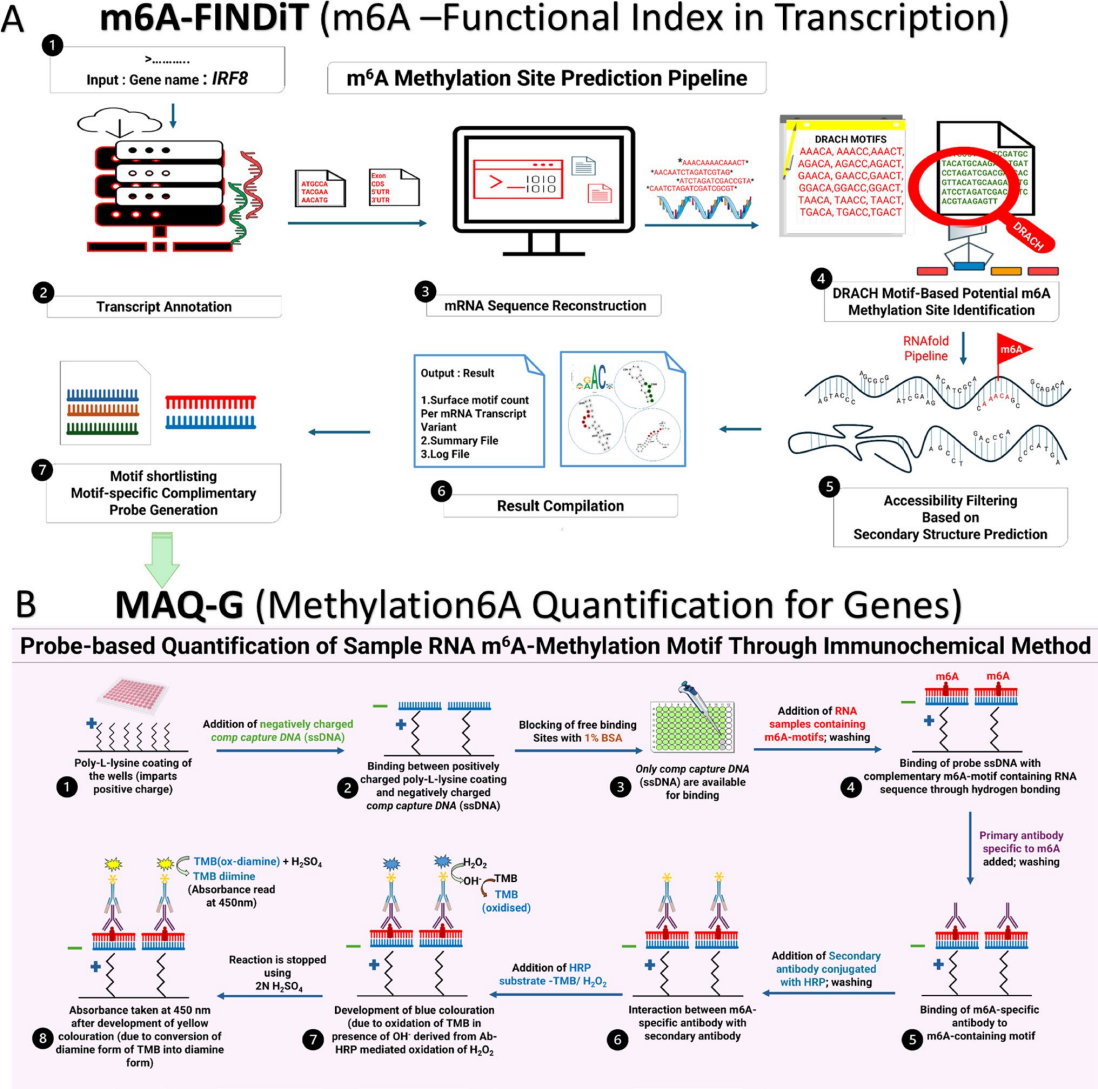
Comprehensive workflow for the identification and quantification of N6-methyladenosine motifs in mRNA transcripts. **(A)** Step-by-step explanation of the m^6^A-methylation site prediction pipeline, m^6^A-FINDiT (m^6^A Functional Index in Transcription). **(B)** Depiction of sequential steps involved in the probe-based detection and quantification of sample RNA m^6^A-methylation motifs through the immunochemical method, MAQ-G (Methylation6A Quantification for Genes)

The pipeline successfully generated a report upon submitting gene names. These computational analysis results were compiled into CSV files for individual transcripts, and a combined dataset was obtained. The files included motif occurrences, motif count, including transcript ID, position, surrounding sequence, and structural context, and summary files of each transcript. A detailed log file containing processing details, such as exon coordinates, sequence lengths, and RNAfold predictions, with ensured transparency and reproducibility, was also recorded. The details of this workflow are depicted in Fig. 1A.

Further validation of the m^6^A modification on the motifs obtained through the computational analysis was performed using a colorimetric method, using custom-made oligonucleotides containing a standard DRACH sequence as motifs with and without the m^6^A modification (Fig. 1B).

### Prediction of m^6^A motifs in *IRF8* mRNA transcript

**Fig. 2:**
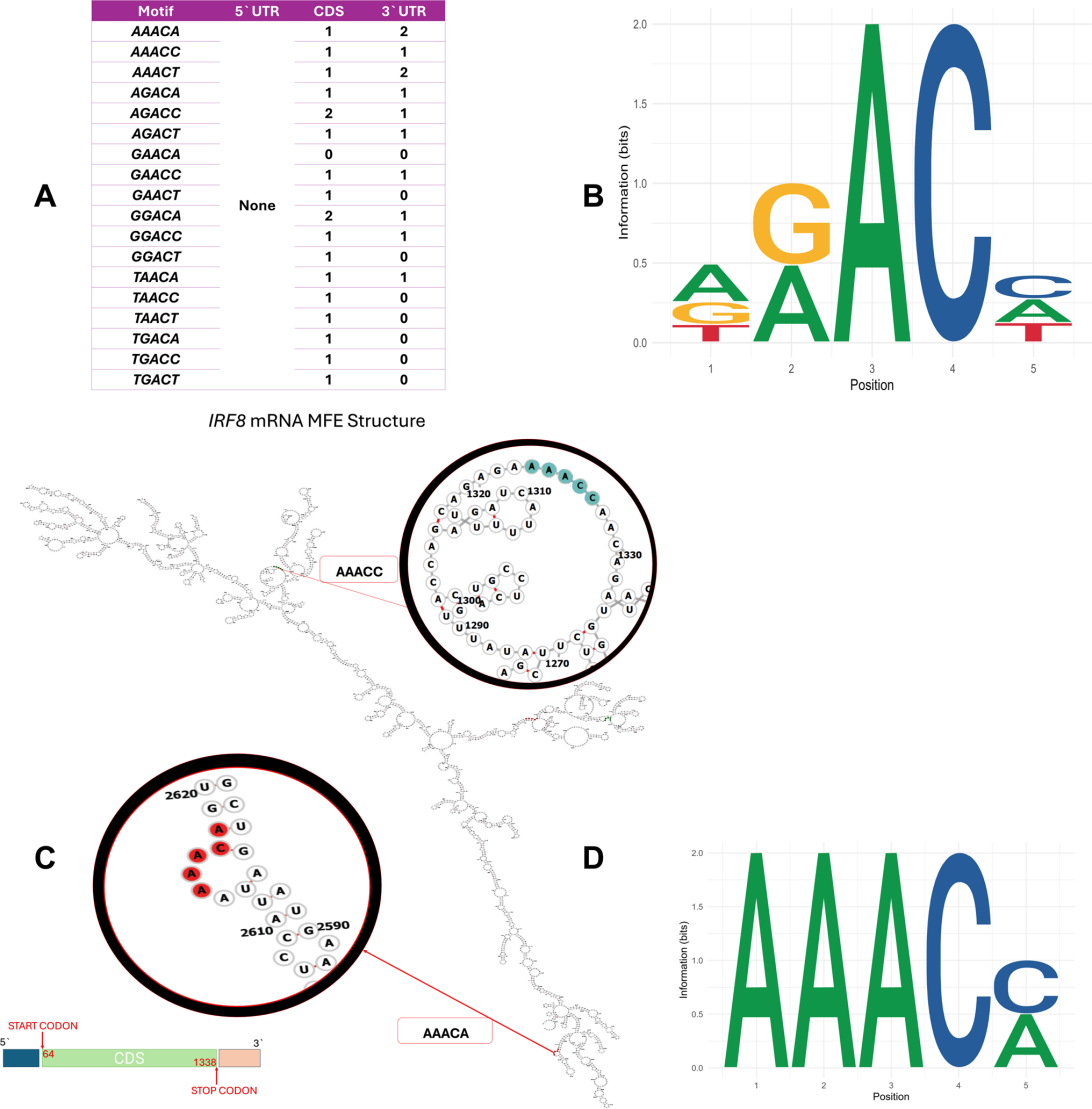
Prediction of m^6^A motifs in *IRF8* mRNA transcript (ENST00000268638.10). **(A)** A tabular representation of 18 unique 5-mer m^6^A motif sequences (DRACH) identified in *IRF8* mRNA, along with their frequencies of occurrence. **(B)** Sequence logo prediction of m6A Motifs in *IRF8* mRNA. The sequence logo illustrates the positional conservation of nucleotides in 5-mer m^6^A motif sequences identified in *IRF8* mRNA. The logo is derived from 18 unique motifs, with frequencies ranging from 0 to 3 occurrences, totaling 31 sequences. Highly conserved positions, such as positions 3 and 4 (adenine and cytosine), are indicated by taller letters, position 2 is dominated by A/G, while more variable positions (e.g., positions 1 and 5) show greater nucleotide diversity. The logo was generated using the ggseqlogo R package. **(C)** *IRF8* mRNA MFE structure prediction (using the Vienna RNA Websuite). This diagram illustrates the predicted minimum free energy (MFE) secondary structure of *IRF8* mRNA. The full MFE structure is shown, with two zoomed-in regions encircled in black, highlighting regions containing m6A motifs AAACC pos 1323–1327 (blue) and AAACA pos 2614–2618 (red) occurring in 3`UTR. **(D)** Sequence logo of the most likely m6A motifs in *IRF8* mRNA based on its secondary structure configuration

Figure 2 provides a comprehensive analysis of m^6^A motifs in *IRF8* mRNA (ENST00000268638.10), elucidating their distribution, sequence conservation, and structural roles. In Fig. 2A, a table identifies 18 unique 5-mer m^6^A motifs (DRACH), including AAACC and AAACA, which are distributed across the CDS and 3’UTR, suggesting potential regulatory roles in these regions. The sequence logo in Fig. 2B, derived from 31 sequences (supplementary Table 2), highlights strong conservation of adenine at position 3 and cytosine at position 4, consistent with the DRACH motif, while positions 1 and 5 exhibit greater nucleotide variability, reflecting flexibility in these positions. Figure 2C presents the predicted minimum free energy (MFE) secondary structure of *IRF8* mRNA, with zoomed-in views revealing m^6^A motifs AAACC (position 1323–1327) and AAACA (position 2614–2618) in the 3’UTR, where their open, unpaired configurations likely facilitate m^6^A modification by enhancing accessibility to writer proteins. Complementing this, the sequence logo of motifs in open conformations, including AAACC and AAACA, underscores their positional conservation, supporting their potential as key m^6^A modification sites that may influence *IRF8* mRNA stability and function (Fig. 2D). Similarly, it was applied to both human and mouse ortholog genes. Total DRACH, accessible DRACH and top preferred motifs are summarized in (Supplementary Table 3).

### MAQ-G technique standardizations

**Fig. 3:**
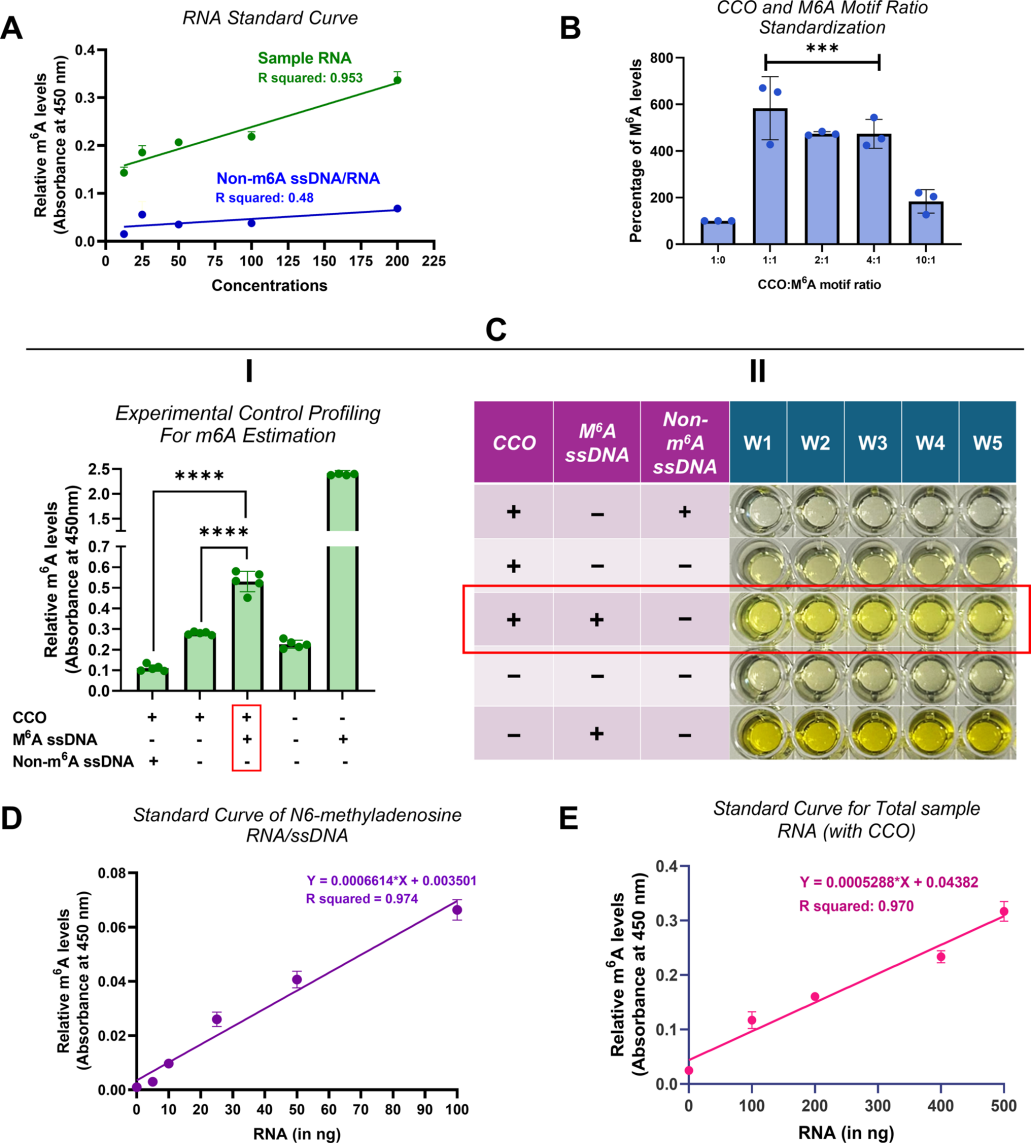
MAQ-G technique standardizations. (**A**) Standardization of the MAQ-G method using increasing concentrations of sample RNA and non-m^6^A methylated ssDNA/RNA. (**B**) Standardization of the ratio of CCO to m^6^A motif checked for assay accuracy and reproducibility. (**C**) Experimental controls standardization for the m^6^A estimation technique. (I) A bar graph depicting the absorbance values of all the experimental controls. (II) 96-well plate image for the controls. The controls were checked in 5 replicates. (**D**) Standard curve depicting the linearity of absorbance values with increasing concentration of N6-methyladenosine RNA/ssDNA. (**E**) Standard curve depicting the linearity of values with increasing concentration of total RNA isolated from testis to a fixed concentration (100ng) of CCO

Firstly, we checked the binding capacity of RNA in 0.01% poly-L-lysine-coated plates and the accuracy of our method for detecting methylation levels. Total RNA was used in increasing concentrations, and methylation levels were checked. With a linear increase in RNA concentration, the normal m^6^A levels detected by the method are expected to increase linearly. We observed a concentration-dependent increase in m^6^A levels with an R^2^ value of 0.953, suggesting a significant association of the values. We also checked for the non-m^6^A ssDNA/RNA at the same concentrations and found that the R^2^ value is 0.48, suggesting a lower correlation between the values (Fig. 3A).

We further wanted to perform m^6^A mRNA motif-specific methylation detection. For this, we used the oligonucleotide sequence, CCO, designed complementary to the m^6^A motif on mRNA (i.e., DRACH). We also designed a DNA oligonucleotide sequence containing the m^6^A motif and the m^6^A modification (referred to as N6A). We standardized the ratio of CCO to N6A. In the case of 1:1, 2:1, and 4:1, we observed high saturation of m^6^A RNA, thereby showing high methylation values. Therefore, we selected a 10:1 ratio of CCO to N6A for further analysis (Fig. 3B).

We experimented with all the necessary controls to verify the effectiveness of our method. Without the addition of poly-L-lysine, the m^6^A methylated RNA binds non-specifically, giving higher absorbance, whereas with the addition of CCO, there is optimal binding and absorbance levels of the complementary m^6^A-methylated RNA. The negative control, i.e., non-methylated DNA (referred to as non-N6A oligos) having the same sequence including the DRACH motif, showed no significant absorbance, confirming the absence of m^6^A modification and no non-specific antibody interaction (Fig. 3C I). The colorimetric representation of the absorbance levels can be observed in the well-plate images of different controls depicted in Fig. 3C II.

Relative m^6^A levels of increasing concentrations of N6-methyladenosine RNA/ssDNA were determined, and a standard curve was generated. The resultant R^2^ value was 0.974, suggesting a strong correlation of the values (Fig. 3D). A similar analysis using increasing concentrations of RNA from biological samples with a fixed concentration of CCO, 100 ng, was performed. The resulting standard curve has an R^2^ value of 0.970, again suggesting a strong correlation of the values (Fig. 3E).

### Validation and m^6^A detection of *IRF8* motifs predicted by computational analysis

The motif-specific m^6^A prediction and validation approach was performed on MOLT-3 cells. *IRF8* was selected as the validation target because a previous study reported that FTO-mediated demethylation regulates *IRF8* expression through specific m^6^A-modified regions within the *IRF8* 3′UTR in MOLT-4 and Jurkat T-ALL cell lines [43]. Using MeRIP-seq, RIP-seq, motif analysis, and MeRIP-qPCR, the authors identified multiple potential m^6^A sites within *IRF8* transcripts and further validated their functional relevance following treatment with the FTO inhibitor FB23-2.

**Fig. 4:**
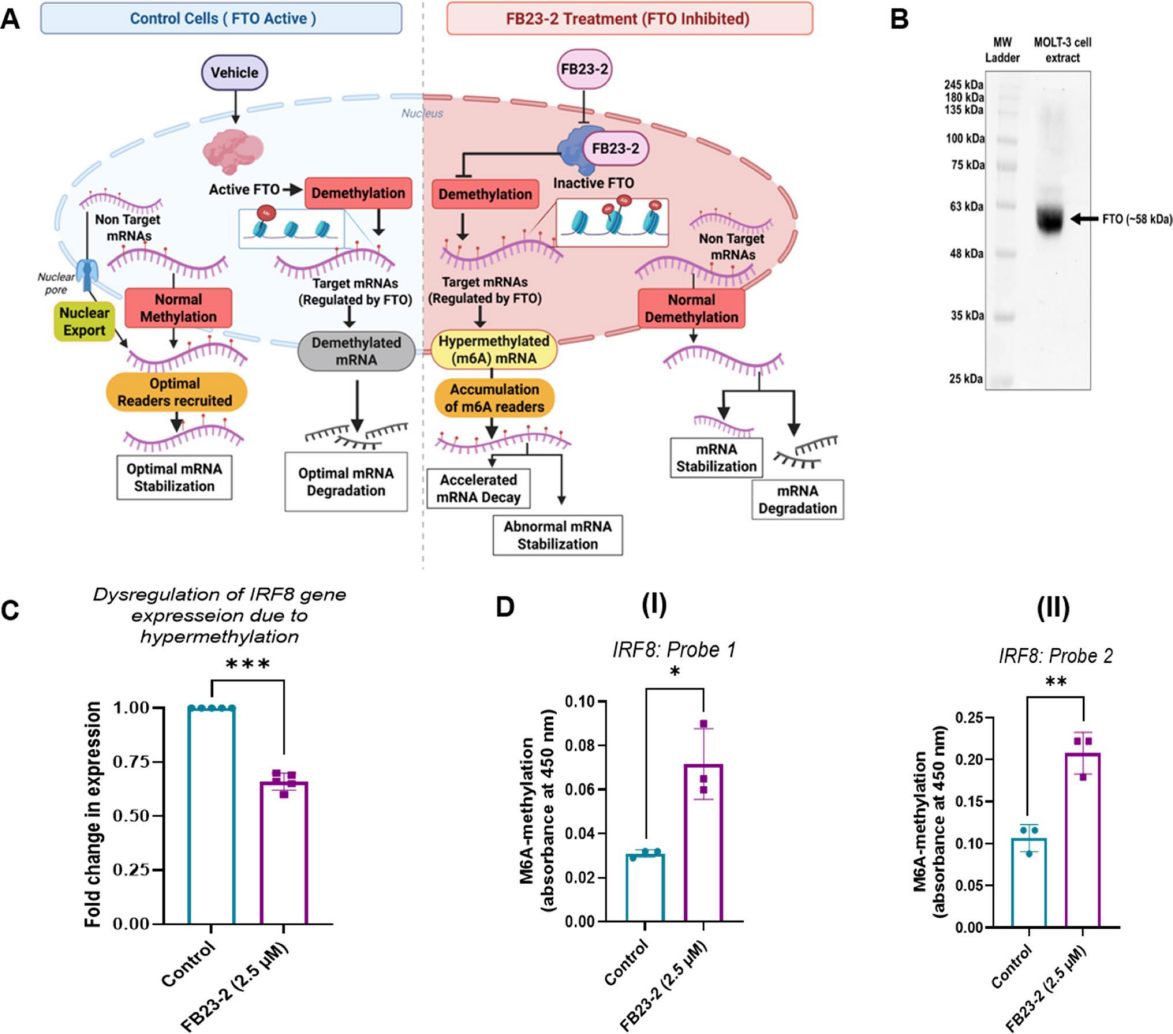
**V**alidation and m6A detection of IRF8 motifs predicted by computational analysis. (**A**) Mechanistic Insight into Transcriptome-Wide m^6^A Fates Under Control and FB23-2 Selective FTO Inhibition Conditions. A. Comparative schematic flow of cellular mRNA fates between vehicle-treated control cells and FB23-2-inhibited cells. (**B**) Western blot image depicting the presence of FTO protein (~ 58 kDa) in MOLT-3 cells. C) Quantitative RT-PCR results to check the expression of *IRF8* in control (vehicle-treated) and FB23-2-treated cells depict a significant decrease in *IRF8* levels in FB23-2-treated cells; qPCR amplicon size is 144 bp. D**)** m^6^A estimation using probes specific to *IRF8* motifs as predicted in the m^6^A-FINDiT. Probes against motifs AGACT (Probe 1) and AAACA (Probe 2) showed significantly increased m^6^A methylation in FB23-2–treated cell RNA (*p* < 0.05 and *p* < 0.01, respectively)

Since these m^6^A-modified regions in *IRF8* had already been experimentally characterised in the same disease context, we selected *IRF8*, which has a tumor suppressor function, as a biologically relevant reference transcript to validate our methodology. Specifically, we applied m^6^A-FINDiT to predict putative m^6^A motifs within *IRF8*. Subsequently, we used our methylation detection kit to experimentally assess methylation changes at these predicted sites. We also performed FB23-2-mediated inhibition of FTO in the MOLT-3 cell line, at a concentration of 2.5 μm for 24 h. Loss of function of FTO caused elevated m^6^A levels in cellular RNA. Figure 4B depicts the presence of FTO in the MOLT-3 cells protein extract. . We further checked the *IRF8* gene expression using qPCR. The *IRF8* mRNA levels were found to be significantly decreased in the FB23-2-treated cells, indicating the hypermethylation-mediated destabilization of *IRF8* mRNA transcripts (Fig. 4C). Finally, to confirm the presence of m^6^A modification at the motif sites predicted by the computational pipeline, we designed CCOs against the two *IRF8* motifs and performed the MAQ-G estimation. We saw a significant increase in the methylation levels at both the motif sites (Fig. 4D).

### Functional validation of predicted m^6^A motifs across multiple cancer types and species

To further establish the functional relevance and broader applicability of our approach, comparative analyses were performed between two acute lymphoblastic leukaemia (ALL) cell lines derived from different species, as well as across two distinct cancer types, to evaluate the conservation and context-dependent variation of predicted m^6^A methylation patterns. This was done to assess the reproducibility and translational relevance of our computational prediction and methylation detection workflow across diverse biological systems.

**Fig. 5:**
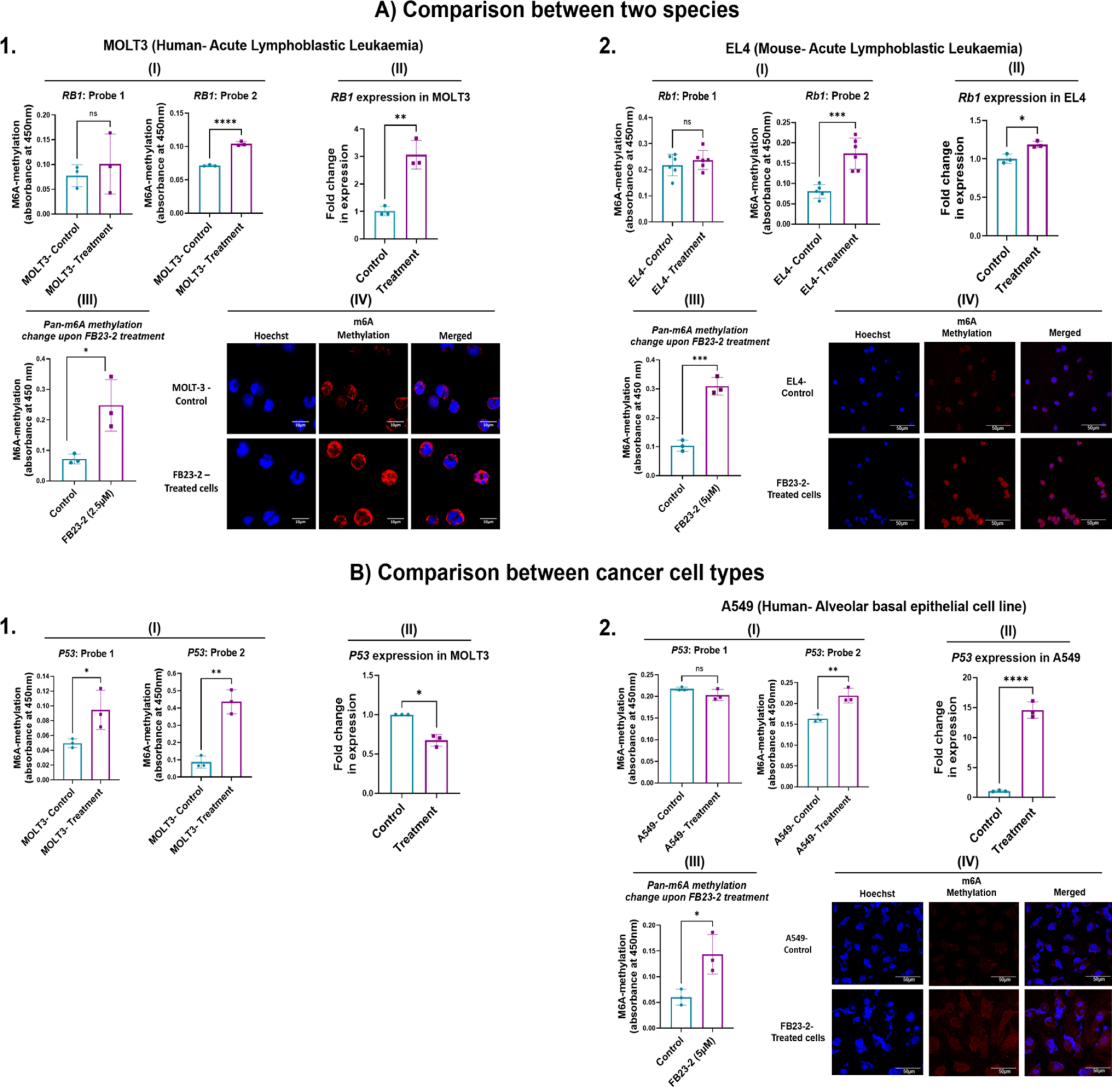
Functional validation of predicted m^6^A motifs across multiple cancer types and species. (**A**) Cross-species validation of computationally predicted RB1-associated m^6^A motifs in human MOLT-3 and murine EL4 T-ALL cell lines following FTO inhibition using FB23-2. (A-1) Validation in MOLT-3 cells. (I) Motif-specific methylation analysis of two predicted RB1-associated DRACH motifs following FB23-2 treatment. Probe 1 did not show a significant increase in methylation, whereas Probe 2 demonstrated significantly increased methylation levels. (II) qPCR analysis showing significant upregulation of *RB1* transcript expression in treated cells. (III) Pan-m^6^A methylation analysis of total RNA demonstrating increased global m^6^A levels following FB23-2 treatment. (IV) Representative confocal immunofluorescence images showing increased cellular m^6^A signal intensity after FB23-2 treatment. (A-2) Validation in EL4 cells. (I) Motif-specific methylation analysis of predicted *Rb1*-associated DRACH motifs following FB23-2 treatment. Probe 1 did not exhibit significant methylation changes, whereas Probe 2 showed significantly increased methylation levels. (II) qPCR analysis showing significant upregulation of *Rb1* transcript expression following treatment. (III) Pan-m^6^A methylation analysis of total RNA showing increased global methylation levels after FTO inhibition. (IV) Representative confocal immunofluorescence images demonstrating increased m^6^A signal intensity in treated cells. (**B**) Cross-cancer validation of computationally predicted *p53*-associated m^6^A motifs in MOLT-3 (human T-ALL) and A549 (human alveolar basal epithelial carcinoma) cell lines following FB23-2 treatment (B-1) Validation in MOLT-3 cells. (I) Motif-specific methylation analysis showing significant increases in methylation levels for predicted *p53*-associated probes following FTO inhibition. (II) qPCR analysis demonstrating significant downregulation of *p53* transcript expression after treatment. (B-2) Validation in A549 cells. (I) Motif-specific methylation analysis showing no significant change in Probe 1 methylation, whereas Probe 2 exhibited significantly increased methylation following FB23-2 treatment. (II) qPCR analysis demonstrating significant upregulation of *p53* transcript expression in treated cells. (III) Pan-m^6^A methylation analysis of total RNA showing increased global methylation levels following treatment. (IV) Representative confocal immunofluorescence images demonstrating increased m^6^A signal intensity in treated cells. Data are represented as mean ± SD from independent biological replicates. Statistical significance was determined using unpaired Student’s t-test. *p < 0.05, **p < 0.01, ***p < 0.001

For cross-species validation within the same cancer type, we utilised two T-ALL cell lines: human MOLT-3 cells and murine EL4 cells (Fig. 5A). RB1, a well-established tumor suppressor gene, was selected for this analysis. Using m^6^A-FINDiT, two putative DRACH consensus motifs with potential regulatory m^6^A methylation sites were identified within the RB1 transcript in both cell lines. Based on these predicted motifs, oligonucleotide probes were designed for motif-specific methylation detection following FTO inhibition using FB23-2.

In MOLT-3 cells, methylation analysis revealed that Probe 1 did not exhibit a significant increase in methylation following FTO inhibition, suggesting that this site may not be directly regulated through FTO-mediated demethylation (Fig. 5A-1I). In contrast, Probe 2 demonstrated a significant increase in methylation levels upon FB23-2 treatment, indicating that the identified motif may function as a potential FTO substrate. qPCR analysis further demonstrated significant upregulation of RB1 transcript levels in treated cells, suggesting enhanced transcript stability following increased methylation (Fig. 5A-1II). Consistently, pan-m^6^A methylation detection of total RNA showed a significant increase in overall methylation levels after FB23-2 treatment (Fig. 5A-1III), which was further supported by increased m^6^A immunofluorescence signal intensity observed in confocal imaging analysis (Fig. 5A-1IV; Supplementary Figure I).

Similarly, in EL4 cells, Probe 1 did not show a significant alteration in methylation levels following FB23-2 treatment, whereas Probe 2 demonstrated a significant increase in methylation (Fig. 5A-2I). qPCR analysis showed significant upregulation of *Rb1* expression in treated EL4 cells (Fig. 5A-2II). Furthermore, pan-m^6^A methylation analysis demonstrated a global increase in methylation levels following FTO inhibition (Fig. 5A-2III), which was also confirmed by enhanced m^6^A immunofluorescence staining in confocal imaging experiments (Fig. 5A-2IV). Collectively, these findings demonstrate that m^6^A-FINDiT and MAQ-G techniques were able to identify transcript-specific m^6^A methylation changes following FTO inhibition across multiple experimental models.

To further assess the applicability of the workflow across distinct cancer types, comparative analyses were performed using MOLT-3 (human T-ALL) and A549 (human alveolar basal epithelial carcinoma) cell lines (Fig. 5B). For this analysis, *TP53*/*p53*, a broadly studied tumor suppressor gene implicated across multiple cancer types, was selected. Using m^6^A-FINDiT, two putative DRACH motifs were identified, and corresponding motif-specific probes were designed for methylation analysis.

In MOLT-3 cells, both predicted *TP53*-associated motifs demonstrated significant increases in methylation following FB23-2 treatment (Fig. 5B-1I). Interestingly, qPCR analysis revealed significant downregulation of *TP53* transcript levels in treated cells (Fig. 5B-1II), suggesting that increased methylation may differentially influence transcript stability depending on cellular context and transcript-specific regulatory mechanisms.

In contrast, analysis in A549 cells showed that Probe 1 did not exhibit a significant change in methylation levels, whereas Probe 2 demonstrated a significant increase in methylation following FTO inhibition (Fig. 5B-2I). qPCR analysis revealed significant upregulation of *TP53* expression in treated A549 cells (Fig. 5B-2II). Additionally, pan-m^6^A methylation analysis showed a significant increase in total methylation levels after FB23-2 treatment (Fig. 5B-2III), which was further supported by enhanced m^6^A immunofluorescence staining observed through confocal imaging (Fig. 5B-2IV). These results demonstrate that m^6^A-FINDiT and MAQ-G can reproducibly identify transcript-specific and context-dependent m^6^A methylation patterns across different species and cancer types.

## Discussion

The *structure-aware* approach of our developed m^6^A-motif finder pipeline is grounded in experimental evidence that m^6^A modifications are enriched in single-stranded regions, as demonstrated by high-resolution mapping techniques [12]. It filters DRACH motifs based on their accessibility, aligning with the biological constraints of m^6^A modification. This work aims to provide a precise and versatile framework for RNA modification research, contributing to a deeper understanding of m^6^A-mediated gene regulation in various biological contexts. Existing m^6^A-methylation prediction tools have only partially met the aforementioned requirements [12, 44]. The likely influence of methylation on the conformation and splicing of mRNA transcripts must be considered in future studies [45, 46]. There remains potential for enhancement, as RNA-protein docking and dynamic simulation data have yet to be integrated into our pipeline to better align these predictions with the physiological state.

To validate the experimental applicability of the workflow, we chose the canonical mRNA transcripts of *IRF8*,* RB1* and *TP53* genes known to play tumor suppressor roles in various kinds of cancers. Previous studies demonstrated that silencing of *IRF8* mediated by m^6^A modification promotes the progression of T-cell acute lymphoblastic leukemia [43, 47]. Using this existing experimental evidence, we validated the reliability and applicability of m6A-FINDiT and the MAQ-G workflow in identifying transcript-specific m6A methylation changes. The use of complementary capture oligonucleotides (CCOs) designed against computationally predicted motifs enabled targeted methylation detection and direct experimental validation of predicted m^6^A sites. This not only enhances the specificity and sensitivity of m^6^A detection but also provides a direct means to validate our computational predictions with experimental evidence. It also ensures robust detection and quantification of m^6^A modifications. Furthermore, this approach utilized readily available and cost-effective reagents, reducing dependency on proprietary components.

Disorders like neurodegeneration, cardiovascular ailments, and cancers require early diagnosis for successful treatments. Genetic mutational markers are often diagnosed from blood or oral DNA samples, and hence they don’t give pictures of what is happening inside different tissues in our body. On the other hand, a tissue biopsy is done only when an individual shows symptoms of disease with altered protein expression or activities. In between these two phases comes the post-transcriptional regulation of various genes, their stability-degradation dynamics, their translatability, and translational efficiency into functional proteins. But these mRNA transcripts are often overlooked for disease diagnosis or treatment measures. This newly developed technique duo should assist in identifying m^6^A motifs that contribute to the stabilisation-degradation dynamics of genes critical for cellular functions such as tumor suppression and anti-inflammatory responses, as well as the hyperstabilization of mRNAs associated with pathological activities like neoplastic growth. Thus, the m^6^A-FINDiT and MAQ-G combination ensures reliable and reproducible results while enhancing the assay accuracy and precision for a wide range of research applications in epitranscriptomics and disease diagnostics.

## Conclusion

In this study, we developed an integrated approach combining a structure-aware computational pipeline (m^6^A-FINDiT) with a probe-based ELISA method (MAQ-G) for the identification and quantification of accessible N6-methyladenosine (m^6^A) motifs in mRNA transcripts. m^6^A-FINDiT effectively predicts DRACH motifs in unpaired regions of mRNA secondary structures, while MAQ-G enables motif-specific, cost-effective experimental validation using complementary capture oligonucleotides. Application of this workflow to tumor suppressor genes (*IRF8*, *RB1*, and *TP53*) across human and mouse cell lines demonstrated its ability to identify functional m^6^A sites, correlate methylation status with transcript stability upon FTO inhibition, and reveal context-dependent effects across cancer types and species.

This dual computational-experimental framework addresses key limitations of existing m^6^A prediction tools by incorporating structural accessibility and providing direct wet-lab integration. The methods hold promise for advancing epitranscriptomic research and may facilitate the development of targeted diagnostic tools for m^6^A-related dysregulation in cancer and other diseases. Future work will focus on expanding validation to clinical samples, incorporating dynamic RNA structure modelling, and evaluating therapeutic potential of motif-specific targeting.

## Supplementary Information

Below is the link to the electronic supplementary material.

Supplementary Material 1: Figure 1-Immunofluorescence of secondary antibody-only control for m⁶A staining in MOLT-3 cells.

Supplementary Material 2: Table 1-Systematic comparison of m^6^A tools.

Supplementary Material 3: Table 2-*IRF8* motif frequency for all transcript variants in tabular format.

Supplementary Material 4: Table 3-Accessibility and preference table.

## Data Availability

No datasets were generated or analysed during the current study.
