# Supplementary figures and images for: A novel method for the identification and quantification of N6-methyladenosine motifs in RNA transcripts
Source: Mol Biol Rep. 2026 Jul 8;53(1):1119. doi: 10.1007/s11033-026-12270-3 (PMC13346147; doi:10.1007/s11033-026-12270-3)

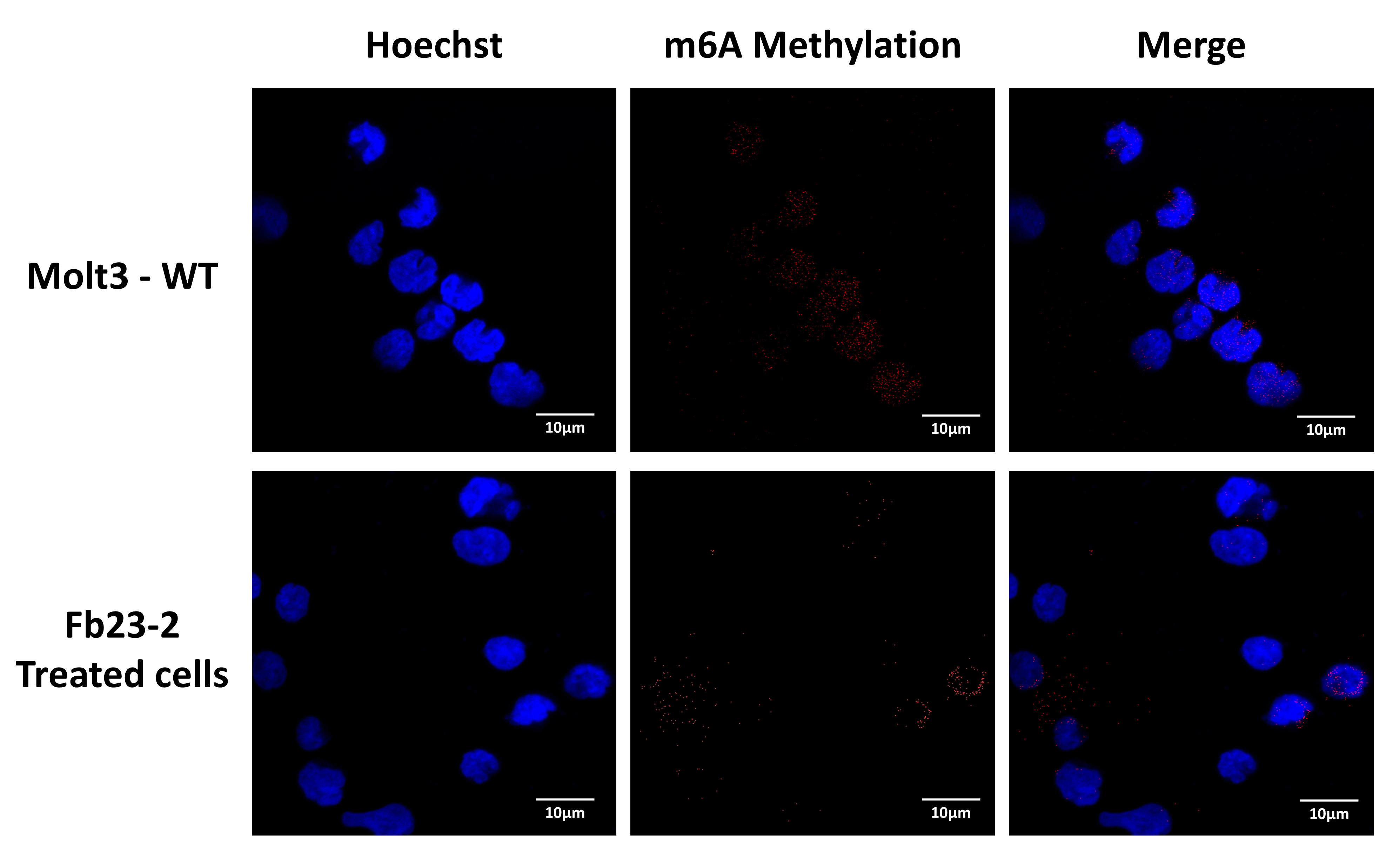

Supplement: Supplementary file 1 — Supplementary Material 1: Figure 1-Immunofluorescence of secondary antibody-only control for m⁶A staining in MOLT-3 cells. [file 11033_2026_12270_MOESM1_ESM.tif]
