# Supplementary material for: A novel method for the identification and quantification of N6-methyladenosine motifs in RNA transcripts
Source: Mol Biol Rep. 2026 Jul 8;53(1):1119. doi: 10.1007/s11033-026-12270-3 (PMC13346147; doi:10.1007/s11033-026-12270-3)
Supplement: Supplementary file 2 — Supplementary Material 2: Table 1-Systematic comparison of m6A tools. [file 11033_2026_12270_MOESM2_ESM.docx]

**Table summarizing features of different m6A tools**

| **Tool** | **Approach / Algorithm** | **Key Inputs / Features** | **RNA Secondary Structure / Accessibility Incorporation** | **Scope (Species / Genes)** | **Main Outputs** | **Experimental / Orthogonal Validation** | **System Requirements & Computational Speed** | **Output Usability & Experimental Integration** | **Key Strengths** | **Reference** |
| --- | --- | --- | --- | --- | --- | --- | --- | --- | --- | --- |
| **Our Pipeline m6A-FINDiT** | Rule-based DRACH scanning + RNAfold MFE | Transcript sequence (GENCODE)  predicted secondary structure | Explicit filter: RNAfold MFE used to retain only unpaired accessible DRACH motifs | Human and Mouse orthologs checked in current study.  Extensible to any gene/species available in GENCODE | DRACH motif list, positions, score, region (CDS/UTR), accessibility status, conservation windows, CCO list | Direct motif-specific validation: Patented probe-based ELISA using CCOs designed from predicted motifs | Low: Standard desktop/laptop.  Python 3 + pandas + ViennaRNA/RNAfold. Seconds–minutes per transcript.  No GPU or training needed. | High usability: Gene-specific CSV outputs with exact motifs + flanking windows; direct integration with patented motif-specific probe-based ELISA | Simple, interpretable, biologically grounded (accessibility filter), lightweight, seamless wet-lab integration. | Current Study |
| **SRAMP** | Random Forest Algorithm | Sequence-derived features (positional nucleotide, KNN, spectrum) | Yes, Incorporates RNAfold-predicted secondary structure | Mammalian  (human + mouse) | Probabilistic m⁶A site scores (full transcript & mature mRNA) | Benchmarked only on MeRIP-seq / miCLIP datasets | Low to Moderate: Web server or R + Perl.  Fast for single sequences. | Probabilistic scores via web server. No direct experimental integration. | Pioneering sequence-only tool | (Zhou et al., 2016) |
| **DeepM6ASeq** | Deep Learning (CNN + BLSTM) | Sequence (one-hot encoding) | CNN learns sequence patterns only | Human, Mouse, Zebrafish | m⁶A site prediction + motif characterization | Benchmarked on miCLIP-seq datasets; saliency maps for interpretation | High: Deep learning framework (GPU beneficial).  Slower for large datasets. | Site predictions + saliency maps for motif visualization. No experimental integration. | Good at motif discovery and biological characterization | (Zhang & Hamada, 2018) |
| **WHISTLE** | Machine Learning (SVM / Random Forest) | Sequence features + 35 genomic features | Yes, Includes RNAfold-predicted secondary structure as part of other genomic features | Human  (multiple cell lines) | High-accuracy transcriptome-wide m⁶A map | Benchmarked on independent MeRIP-seq / miCLIP datasets | Moderate to High: Extensive genomic feature extraction. Suitable for transcriptome-wide runs. | Transcriptome-wide map via web server. No motif-specific experimental integration. | High accuracy using rich genomic context | (K. Chen et al., 2019) |
| **HSM6AP** | XGBoost with multiple weights & feature stitching | Sequence-derived + gene-derived features (23 features) | No | Human | High-precision m⁶A site prediction | Benchmarked on independent test sets | Moderate to High: XGBoost (Python/R). Efficient once trained. | High-precision predictions. No experimental integration mentioned. | Sample weighting + feature fusion improves performance over WHISTLE | (Li et al., 2021) |
| **m6Aboost** | Machine Learning model  (m6Aboost) | Experimental miCLIP2 features + RNA sequence features | Focuses on miCLIP2 data | Human and mouse cell lines | m⁶A site prediction in miCLIP2 data | Calibrated on Mettl3-knockout miCLIP2 data; orthogonal validation with orthogonal methods | Moderate: Trained ML model integrated with miCLIP2 pipeline. | High-confidence sites tailored for miCLIP2 experimental data. | Addresses false positives in antibody-based data | (Körtel et al., 2021) |
| **DeepM6ASeq-EL** | Deep Learning (LSTM + CNN ensemble, hard voting) | Sequence encoding (one-hot, word2vec.) | Sequence encoding only | Human cell line | m⁶A site prediction (full transcript & mature mRNA) | Benchmarked against WHISTLE on independent datasets | High: DL libraries (PyTorch/TensorFlow), GPU beneficial. | Site predictions.  No direct experimental integration. | Ensemble improves robustness; tested against WHISTLE | (J. Chen et al., 2022) |
| **CLSM6A** | Deep Learning (CNN-based) | Sequence (ENAC encoding) | ENAC encoding only | Human  (Cell lines + Tissues) | Cell-line/tissue-specific m⁶A prediction + interpretable motifs | Benchmarked on well-curated cell-line/tissue datasets | High: CNN deep learning model. | Interpretable motifs + saliency maps. No motif-specific experimental integration. | Cell-line specific; interpretable via motifs and saliency maps | (Zhang et al., 2023) |
| **Deep**  **SRAMP** | Hybrid Deep Learning (Transformer + BiGRU) | Sequence features + genomic features | Sequence + genomic features only | Mammalian | High-accuracy m⁶A site prediction | Benchmarked on multiple datasets (strong generalization) | High: Transformer + RNN (GPU recommended). | High-accuracy predictions including isoform level. No direct experimental integration. | State-of-the-art performance; strong generalization | (Fan et al., 2024) |

**Reference**

Chen, J., Zou, Q., & Li, J. (2022). DeepM6ASeq-EL: Prediction of human N6-methyladenosine (m6A) sites with LSTM and ensemble learning. *Frontiers of Computer Science*, *16*(2), 162302. https://doi.org/10.1007/s11704-020-0180-0

Chen, K., Wei, Z., Zhang, Q., Wu, X., Rong, R., Lu, Z., Su, J., de Magalhães, J. P., Rigden, D. J., & Meng, J. (2019). WHISTLE: A high-accuracy map of the human N6-methyladenosine (m6A) epitranscriptome predicted using a machine learning approach. *Nucleic Acids Research*, *47*(7), e41–e41. https://doi.org/10.1093/nar/gkz074

Fan, R., Cui, C., Kang, B., Chang, Z., Wang, G., & Cui, Q. (2024). A combined deep learning framework for mammalian m6A site prediction. *Cell Genomics*, *4*(12), 100697. https://doi.org/10.1016/j.xgen.2024.100697

Körtel, N., Rücklé, C., Zhou, Y., Busch, A., Hoch-Kraft, P., Sutandy, F. X. R., Haase, J., Pradhan, M., Musheev, M., Ostareck, D., Ostareck-Lederer, A., Dieterich, C., Hüttelmaier, S., Niehrs, C., Rausch, O., Dominissini, D., König, J., & Zarnack, K. (2021). Deep and accurate detection of m6A RNA modifications using miCLIP2 and m6Aboost machine learning. *Nucleic Acids Research*, *49*(16), e92–e92. https://doi.org/10.1093/nar/gkab485

Li, J., He, S., Guo, F., & Zou, Q. (2021). HSM6AP: A high-precision predictor for the Homo *sapiens* N6-methyladenosine (m^6 A) based on multiple weights and feature stitching. *RNA Biology*, *18*(11), 1882–1892. https://doi.org/10.1080/15476286.2021.1875180

Zhang, Y., & Hamada, M. (2018). DeepM6ASeq: Prediction and characterization of m6A-containing sequences using deep learning. *BMC Bioinformatics*, *19*(S19), 524. https://doi.org/10.1186/s12859-018-2516-4

Zhang, Y., Wang, Z., Zhang, Y., Li, S., Guo, Y., Song, J., & Yu, D.-J. (2023). Interpretable prediction models for widespread m6A RNA modification across cell lines and tissues. *Bioinformatics*, *39*(12), btad709. https://doi.org/10.1093/bioinformatics/btad709

Zhou, Y., Zeng, P., Li, Y.-H., Zhang, Z., & Cui, Q. (2016). SRAMP: Prediction of mammalian N^6^ -methyladenosine (m^6^ A) sites based on sequence-derived features. *Nucleic Acids Research*, *44*(10), e91–e91. https://doi.org/10.1093/nar/gkw104
