# Supplementary material for: A novel method for the identification and quantification of N6-methyladenosine motifs in RNA transcripts
Source: Mol Biol Rep. 2026 Jul 8;53(1):1119. doi: 10.1007/s11033-026-12270-3 (PMC13346147; doi:10.1007/s11033-026-12270-3)
Supplement: Supplementary file 3 — Supplementary Material 3: Table 2-IRF8 motif frequency for all transcript variants in tabular format. [file 11033_2026_12270_MOESM3_ESM.docx]

| Motif | ENST00000268638.10 | ENST00000562492.5 | ENST00000563180.1 | ENST00000564056.1 | ENST00000564617.5 | ENST00000564803.6 | ENST00000565552.1 | ENST00000566369.2 | ENST00000569145.5 | ENST00000569607.2 | ENST00000570088.1 | ENST00000696884.1 | ENST00000696885.1 | ENST00000696886.1 | ENST00000696887.1 | ENST00000696888.1 | ENST00000696889.1 | ENST00000696890.1 |  |
| --- | --- | --- | --- | --- | --- | --- | --- | --- | --- | --- | --- | --- | --- | --- | --- | --- | --- | --- | --- |
| AAACA | 3 | 0 | 3 | 1 | 1 | 3 | 2 | 3 | 0 | 3 | 2 | 3 | 6 | 8 | 3 | 2 | 1 | 2 |  |
| AAACC | 2 | 1 | 3 | 0 | 0 | 2 | 0 | 2 | 0 | 2 | 0 | 2 | 4 | 6 | 2 | 0 | 0 | 3 |  |
| AAACT | 3 | 1 | 0 | 1 | 1 | 3 | 1 | 3 | 0 | 2 | 0 | 3 | 4 | 7 | 3 | 3 | 3 | 3 |  |
| AGACA | 2 | 0 | 3 | 0 | 0 | 2 | 0 | 2 | 0 | 2 | 1 | 2 | 2 | 3 | 2 | 1 | 0 | 2 |  |
| AGACC | 3 | 1 | 0 | 0 | 0 | 3 | 0 | 3 | 0 | 3 | 2 | 3 | 3 | 5 | 3 | 0 | 0 | 3 |  |
| AGACT | 2 | 0 | 0 | 1 | 0 | 2 | 0 | 1 | 0 | 1 | 0 | 1 | 2 | 3 | 2 | 0 | 0 | 2 |  |
| GAACA | 0 | 1 | 1 | 0 | 0 | 0 | 0 | 0 | 1 | 1 | 0 | 0 | 1 | 1 | 0 | 2 | 2 | 0 |  |
| GAACC | 2 | 0 | 1 | 1 | 1 | 2 | 0 | 2 | 0 | 2 | 0 | 2 | 3 | 4 | 2 | 1 | 1 | 2 |  |
| GAACT | 1 | 0 | 2 | 0 | 0 | 1 | 1 | 1 | 0 | 1 | 0 | 1 | 2 | 2 | 1 | 0 | 0 | 1 |  |
| GGACA | 3 | 0 | 4 | 2 | 2 | 3 | 0 | 3 | 0 | 1 | 0 | 3 | 6 | 8 | 3 | 2 | 3 | 3 |  |
| GGACC | 2 | 0 | 1 | 1 | 1 | 2 | 0 | 2 | 0 | 1 | 0 | 2 | 2 | 2 | 2 | 1 | 1 | 2 |  |
| GGACT | 1 | 0 | 1 | 0 | 1 | 1 | 1 | 1 | 0 | 1 | 2 | 1 | 1 | 1 | 1 | 1 | 1 | 0 |  |
| TAACA | 2 | 1 | 0 | 0 | 0 | 2 | 0 | 2 | 0 | 2 | 0 | 2 | 3 | 3 | 2 | 0 | 1 | 2 |  |
| TAACC | 1 | 0 | 0 | 0 | 0 | 1 | 0 | 1 | 0 | 1 | 0 | 1 | 2 | 4 | 1 | 0 | 1 | 1 |  |
| TAACT | 1 | 0 | 1 | 0 | 0 | 1 | 1 | 1 | 0 | 1 | 0 | 1 | 2 | 3 | 1 | 0 | 0 | 1 |  |
| TGACA | 1 | 0 | 2 | 0 | 1 | 1 | 1 | 1 | 0 | 1 | 2 | 1 | 2 | 2 | 1 | 2 | 1 | 0 |  |
| TGACC | 1 | 0 | 4 | 0 | 1 | 1 | 1 | 1 | 0 | 0 | 1 | 1 | 2 | 5 | 1 | 1 | 0 | 0 |  |
| TGACT | 1 | 0 | 0 | 0 | 0 | 1 | 0 | 1 | 0 | 1 | 0 | 1 | 2 | 4 | 1 | 2 | 0 | 2 |  |
