# Supplementary material for: A novel method for the identification and quantification of N6-methyladenosine motifs in RNA transcripts
Source: Mol Biol Rep. 2026 Jul 8;53(1):1119. doi: 10.1007/s11033-026-12270-3 (PMC13346147; doi:10.1007/s11033-026-12270-3)
Supplement: Supplementary file 4 — Supplementary Material 4: Table 3-Accessibility and preference table. [file 11033_2026_12270_MOESM4_ESM.docx]

| Gene | Species | Total_DRACH | Accessible_DRACH | Percent_Accessible | Top_3_Preferred_Motifs |
| --- | --- | --- | --- | --- | --- |
| IRF8 | Human | 70 | 33 | 47.14 | GAACC (4); AAACC (4); TAACC (4) |
| Irf8 | Mouse | 65 | 37 | 56.92 | GAACC (6); TGACT (5); TGACC (4) |
| TP53 | Human | 45 | 22 | 48.89 | AGACT (4); AAACC (4); AAACT (3) |
| Trp53 | Mouse | 61 | 32 | 52.46 | AAACT (5); AGACT (4); AGACA (3) |
| RB1 | Human | 103 | 50 | 48.54 | AAACA (10); AAACT (8); GGACC (4) |
| RB1 | Mouse | 91 | 52 | 57.14 | AAACA (10); AAACT (6); TGACA (5) |

**m6A-FINDiT output**

Accessibility and preference table
